# Anti-Inflammatory and Anti-Oxidative Activity of Indole-3-Acetic Acid Involves Induction of HO-1 and Neutralization of Free Radicals in RAW264.7 Cells

**DOI:** 10.3390/ijms21051579

**Published:** 2020-02-25

**Authors:** Yun Ji, Wenzhen Yin, Yuan Liang, Lijun Sun, Yue Yin, Weizhen Zhang

**Affiliations:** Department of Physiology and Pathophysiology, Peking University Health Science Center, and Key Laboratory of Molecular Cardiovascular Science, Ministry of Education, Beijing 100191, China; jean500@163.com (Y.J.); wenzhenyin@sina.cn (W.Y.); liangyuan@bjmu.edu.cn (Y.L.); sunlj9002@163.com (L.S.); weizhenzhang@bjmu.edu.cn (W.Z.)

**Keywords:** indole-3-acetic acid, inflammation, macrophage, LPS, HO-1, free radical

## Abstract

The cellular and molecular mechanisms by which indole-3-acetic acid (IAA), a tryptophan-derived metabolite from gut microbiota, attenuates inflammation and oxidative stress has not been fully elucidated. The present study was to unearth the protective effect and underlying mechanism of IAA against lipopolysaccharide (LPS)-induced inflammatory response and free radical generation in RAW264.7 macrophages. IAA significantly ameliorated LPS-induced expression of interleukin-1β (IL-1β), interleukin-6 (IL-6), and monocyte chemoattractant protein-1 (MCP-1) as well as generation of reactive oxidative species (ROS) and nitric oxide (NO). LPS-triggered nuclear translocation of nuclear factor kappa B (NF-κB) p65 was mitigated by IAA treatment. Further, an up-regulation of heme oxygenase-1 (HO-1) was observed in IAA-treated cells in dose-dependent manner under both normal and LPS-stimulated condition. Interference of HO-1 activity by tin protoporphyrin IX (SnPP) impeded the alleviative effects of IAA on expression of IL-1β and IL-6 induced by LPS, whereas demonstrated no effect on its suppression of ROS and NO production. This result suggests a HO-1-dependent anti-inflammatory effect of IAA and its direct scavenging action on free radicals. Treatment with CH-223191, a specific antagonist of aryl hydrocarbon receptor (AhR), showed no significant effects on the beneficial role of IAA against inflammation and free radical generation. In summary, our findings indicate that IAA alleviates LPS-elicited inflammatory response and free radical generation in RAW264.7 macrophages by induction of HO-1 and direct neutralization of free radicals, a mechanism independent of AhR.

## 1. Introduction

Gut microbiota dysbiosis has emerged as an interferent of systemic immune homeostasis [1]. Long-term unhealthy diet-induced alteration of intestinal flora may contribute to metabolic endotoxemia characterized by the increase of lipopolysaccharide (LPS) in blood concentration [2]. As the first line of defense in the body against infectious pathogens, macrophages play crucial roles in the progression of tissue inflammation in response to LPS [3]. LPS activates macrophages to secrete proinflammatory cytokines (e.g., tumor necrosis factor-a (TNF-α), interleukin (IL)-1, and IL-6) and free radicals (e.g., reactive oxidative species (ROS) and nitric oxide (NO)) [4,5]. Moderate production of inflammatory cytokines from macrophages effectively inhibits the proliferation and spreading of invading pathogens, whereas excessive inflammatory cytokines and free radicals may lead to tissue damage, disordered organ function, and inflammatory diseases [6,7].

Tryptophan is an essential amino acid that exerts versatile biological function in humans and animals [8,9]. In recent years, much attention has been paid to tryptophan metabolites from gut microbiota such as indole-3-acetic acid (IAA). The topical anti-inflammatory activity of IAA has been demonstrated in the mouse model of ear edema by Jones et al. [10]. The anti-inflammatory role of IAA is probably associated with its anti-oxidative activity. Findings from our previous study revealed that IAA attenuates the progression of nonalcoholic fatty liver disease (NAFLD) via suppression of oxidative and inflammatory stress [11]. Innate immunity mediated by macrophages in response to low-dose LPS plays a major role in the progression of NAFLD [12]. Indeed, highly activated macrophages caused by low-dose LPS have been implicated in the pathogenesis of various chronic inflammatory diseases [13,14]. However, the underlying mechanism by which IAA alleviates inflammatory response in macrophages remains unknown. Herein, we investigated the ability of IAA in cultured macrophages to attenuate the inflammatory response and free radical elicited by LPS. Since heme oxygenase-1 (HO-1) has been recognized as a critical immune regulator in macrophages [15] and IAA was identified as an endogenous AhR ligand, we also explored whether the effect of IAA on inflammatory response and oxidative stress in macrophages exposed to LPS is dependent on HO-1 and/or AhR.

## 2. Results

### 2.1. Effect of IAA on Cell Viability and Morphology of RAW264.7 Cells Treated with LPS

**Figure 1:**
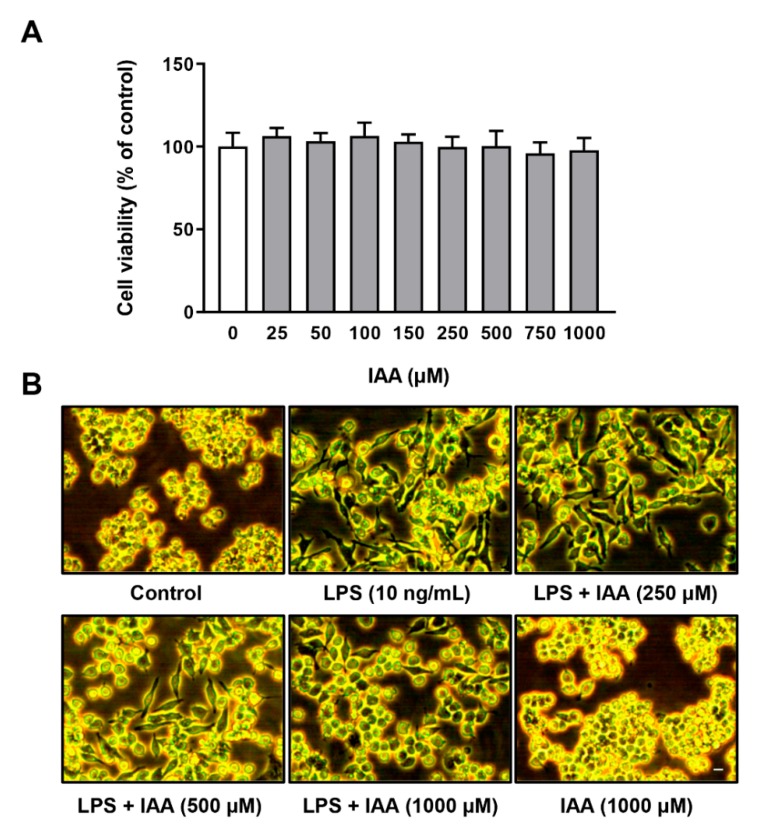
Effects of IAA on cell viability of RAW264.7 macrophages. (**A**) Dose-dependent effect of IAA on cell viability of the RAW264.7 macrophages. Cells were treated with multiple dose of IAA for 28 h in DMEM supplemented with 5% FBS. Cell viability was determined by CCK-8 assay. Values are given as means ± SEM. *n* = 6. (**B**) IAA blocked the morphological alterations of RAW264.7 cells induced by LPS in dose-dependent manner. Cells were pretreated with various concentrations of IAA as indicated for 12 h, followed by LPS (10 ng/mL) exposure for another 12 h with or without IAA. Images were captured using a phase contrast microscope. Scale bar represents 10 μm. IAA, indole-3-acetic acid; LPS, lipopolysaccharide; CCK-8, cell counting kit-8.

The viability of RAW264.7 cells exposed to IAA at various concentrations for 28 h was evaluated by CCK-8 assay. As shown in Figure 1A, IAA at concentration ranging from 25–1000 µM had no effect on cell viability in RAW264.7 cells. The morphological changes of RAW264.7 cells subjected to IAA and LPS treatment were observed under an optical microscopy. Cells were pre-treated with 0, 250, 500, and 1000 µM of IAA for 12 h, followed by treatment with LPS (10 ng/mL) in the presence or absence of IAA for an additional 12 h. The control group of RAW264.7 cells were round in shape without pseudopodia, whereas those incubated with LPS for 12 h showed increase in cell size and became spindle-shaped with elongated pseudopodia. IAA treatment ameliorated the morphological alteration induced by LPS in a dose-dependent manner. No morphological change was found in cells receiving IAA alone treatment (Figure 1B).

### 2.2. Alleviation of LPS-Induced Proinflammatory Response by IAA

**Figure 2:**
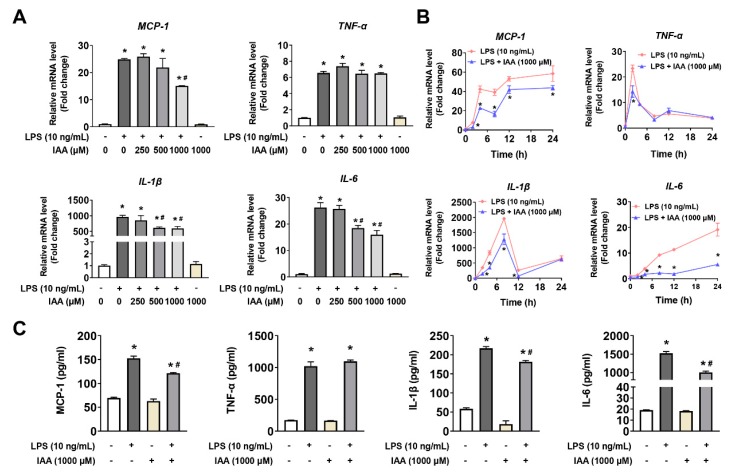
Effects of IAA on LPS-induced mRNA expression and secretion of proinflammatory cytokines in RAW 264.7 cells. (**A**) mRNA levels relative to β-actin in cells exposed to LPS following treatment with IAA. Cells were treated with various concentrations (0, 250, 500, 1000 μM) of IAA for 12 h, and then incubated with LPS (10 ng/mL) in the presence or absence of IAA for 8 h. β-actin was considered as house-keeping gene. Results are mean ± SEM. *n* = 3. * *p* < 0.05 versus control group; ^#^ *p* < 0.05 versus LPS group. (**B**) Time-course results of relative mRNA levels of proinflammatory cytokines. Cells were pretreated with or without IAA for 12 h, followed by LPS stimulation in the present or absence of IAA for 0, 2, 4, 8, 12, and 24 h. β-actin was regarded as reference gene. Results are shown as mean ± SEM. *n* = 3. * *p* < 0.05 versus LPS group. (**C**) Secretion of proinflammatory cytokines were detected by ELISA at 16 h following LPS stimulation in the presence or absence of IAA after a 12 h treatment with IAA. Results shown are mean ± SEM. *n* = 3. * *p* < 0.05 versus control; ^#^ *p* < 0.05 versus LPS group. IAA, indole-3-acetic acid; LPS, lipopolysaccharide.

**Figure 3:**
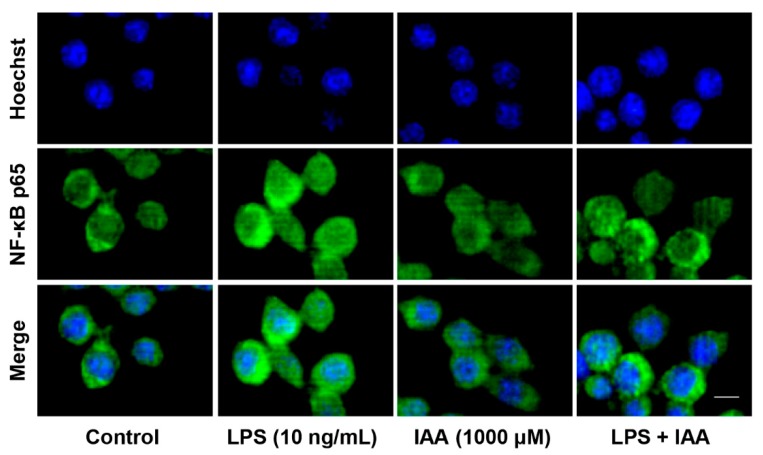
LPS-induced nuclear translocation of NF-κB p65 was alleviated by IAA treatment. Cells were pretreated with IAA (1000 μM) for 12 h before 2 h LPS (10 ng/mL) stimulation, after which NF-κB p65 was determined by immunofluorescence. Scale bar represents 10 μm. IAA, indole-3-acetic acid; LPS, lipopolysaccharide; NF-κB, nuclear factor kappa B.

mRNA and protein levels of proinflammatory cytokines were analyzed by real time PCR and ELISA analysis. As shown in Figure 2A, LPS exposure for 8 h resulted in a significant up-regulation of MCP-1, TNF-α, IL-1β, and IL-6 at transcriptional level compared with the vehicle control (*p* < 0.05). By contrast, 500 and 1000 μM of IAA significantly attenuated LPS-induced increase in IL-1β and IL-6 mRNA levels (*p* < 0.05). The inhibitory effect of IAA on the elevation in MCP-1 mRNA induced by LPS was observed in cells treated with 1000 μM of IAA (*p* < 0.05). Next, we examined the time-course effect of LPS on the mRNA level of MCP-1, TNF-α, IL-1β, and IL-6 in RAW264.7 in the presence or absence of 1000 μM of IAA. As presented in Figure 2B, treatment with IAA attenuated LPS-induced up-regulation of MCP-1 and IL-6 mRNA from 2 to 24 h (*p* < 0.05). The elevation in IL-1β mRNA levels in response to LPS was alleviated within 12 h in the presence of IAA (*p* < 0.05). The inhibitory effect of IAA on the expression of TNF-α mRNA induced by LPS was only observed at 2 h (*p* < 0.05). Consistent with the results at transcriptional level, IAA also blocked the up-regulation in the secretion of proinflammatory cytokines induced by LPS (Figure 2C, *p* < 0.05). LPS significantly increased levels of MCP-1, IL-1β, and IL-6 in supernatants of cultured medium (*p* < 0.05). By contrast, 1000 μM of IAA attenuated the increment of MCP-1, IL-1β, and IL-6 induced by LPS in RAW264.7 macrophages (*p* < 0.05). Since nuclear factor kappa B (NF-κB) is critical for the immune response, we next examined the translocation of the NF-κB p65 subunit by immunofluorescence. As shown in Figure 3, LPS exposure for 1 h induced a nuclear accumulation of p65, which was attenuated visibly by IAA treatment, suggesting that IAA inhibits the activation of NF-κB.

### 2.3. Suppression of Free Radical Production by IAA

**Figure 4:**
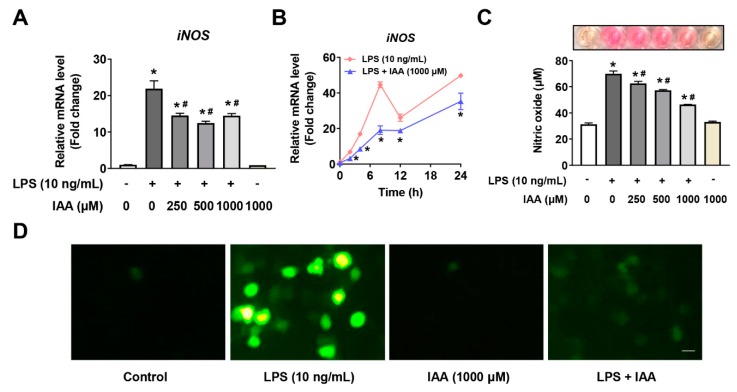
IAA inhibited the generation of NO and ROS induced by LPS in RAW264.7 cells. (**A**) Cells were incubated with various concentrations of IAA followed by LPS (10 ng/mL) for 8 h in the absence or presence IAA for 8 h, after which iNOS mRNA was determined by RT-qPCR. β-actin was used as the reference gene. Results are shown as mean ± SEM. *n* = 3. * *p* < 0.05 versus control group; ^#^ *p* < 0.05 versus LPS group. (**B**) Time-dependent effect of LPS or LPS + IAA on the expression of iNOS mRNA. Data are shown as mean ± SEM. *n* = 3. * *p* < 0.05 versus LPS group. Cells pre-incubated with indicated concentrations of IAA for 12 h prior to an additional 16 h exposure to LPS, after which NO content (**C**) in culture medium and intracellular ROS production (**D**) were evaluated as described in Materials and Methods. Results presents mean ± SEM. *n* = 3. * *p* < 0.05 versus control; ^#^ *p* < 0.05 versus LPS group. Scale bar represents 10 μm. iNOS, inducible nitric oxide synthase; NO, nitric oxide; ROS, reactive oxidative species; IAA, indole-3-acetic acid; LPS, lipopolysaccharide.

To evaluate weather IAA scavenge free radical in RAW264.7 cells subjected to LPS treatment, NO and ROS production were measured. LPS significantly increased the iNOS mRNA expression and NO production in RAW264.7 cells (*p* < 0.05). IAA treatment dose-dependently suppressed LPS-induced iNOS mRNA expression in RAW264.7 cells and NO generation in culture supernatant (Figure 4A,C). This effect occurred from 2 to 24 h (Figure 4B) (*p* < 0.05). Results from 2′,7′-dichlorofluorescin diacetate (DCFH-DA) staining demonstrated that LPS increased the formation of ROS in RAW264.7 cells, which was mostly counteracted by IAA treatment. Treating cells with IAA without LPS did not affect the production of NO or ROS. These evidences suggest that IAA functions to scavenge free radical generated under LPS-induced inflammatory condition in RAW264.7 macrophages.

### 2.4. Increment of HO-1 Levels in RAW 264.7 Cells in the Presence and Absence of LPS

**Figure 5:**
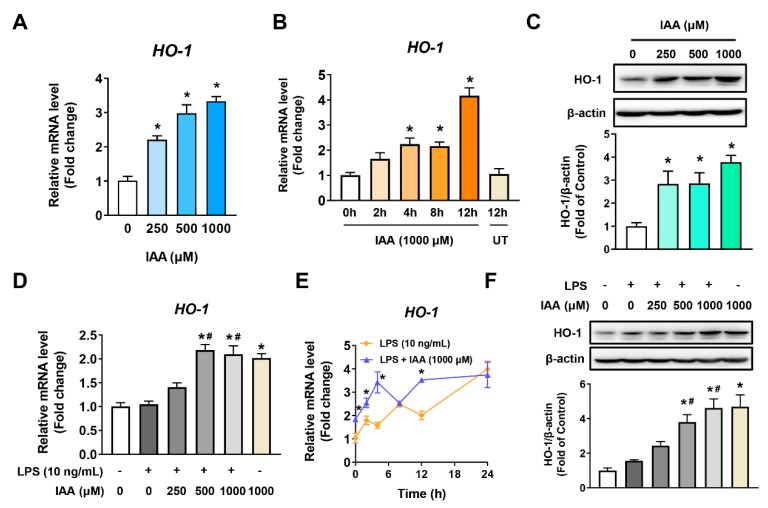
Induction of HO-1 expression in RAW264.7 in response to IAA treatment. Cells were (**A**) treated with different doses of IAA, as shown for 12 h, or (**B**) exposed to 1000 μM of IAA for indicated time points, followed by RT-qPCR analysis for HO-1 mRNA. β-actin was considered as the reference. Results are shown as mean ± SEM. *n* = 3. * *p* < 0.05 versus control group. (**C**) Western blot analysis of HO-1 protein. Cells were incubated with indicated concentrations of IAA for 12 h. β-actin was regarded as the loading control. Bar chart are mean ± SEM. *n* = 3. * *p* < 0.05 versus control group. (**D**) Cell were incubated with medium-containing different concentrations of IAA for 12 h, after which cells were stimulated with LPS for 8 h and then analyzed by RT-qPCR. Values are shown as mean ± SEM. *n* = 3. * *p* < 0.05 versus control group; ^#^ *p* < 0.05 versus LPS group. (**E**) Time-course analysis of HO-1 mRNA in response to LPS or LPS plus IAA treatment. Results are described as mean ± SEM. *n* = 3. * *p* < 0.05 versus LPS group. (**F**) Cells pretreated with IAA for 12 h were subjected to LPS (10 ng/mL) incubation for 16 h before Western blotting. β-actin was considered as the loading control. Results are shown as mean ± SEM. *n* = 3. * *p* < 0.05 versus control group. ^#^ *p* < 0.05 versus LPS group. HO-1, heme oxygenase-1; IAA, indole-3-acetic acid; LPS, lipopolysaccharide.

The mRNA levels of HO-1 were increased in dose depending on the content of IAA and peaked at 12 h in RAW264.7 cells following 1000 μM of IAA treatment (*p* < 0.05) (Figure 5A,B). Likewise, IAA enhanced the expression of HO-1 protein in a concentration-dependent manner (*p* < 0.05) (Figure 5C). To examine whether IAA-induced HO-1 protein expression was effective under the stimulation of LPS, RT-qPCR and Western blot analysis were also performed on cells following LPS treatment in the presence or absence of IAA. As seen in Figure 5D, IAA dose-dependently increased the mRNA abundance of HO-1 in cells treated with LPS. No difference in HO-1 mRNA levels was observed between cells exposed to 1000 μM IAA with or without LPS for various times up to 12 h (*p* < 0.05) (Figure 5E). Western blot analysis further demonstrated that IAA significantly increases HO-1 levels in a dose-dependent manner even in the presence of LPS stimulation (*p* < 0.05) (Figure 5F).

### 2.5. IAA Attenuates the Generation of IL-1β and IL-6 in HO-1-Dependent Manner

**Figure 6:**
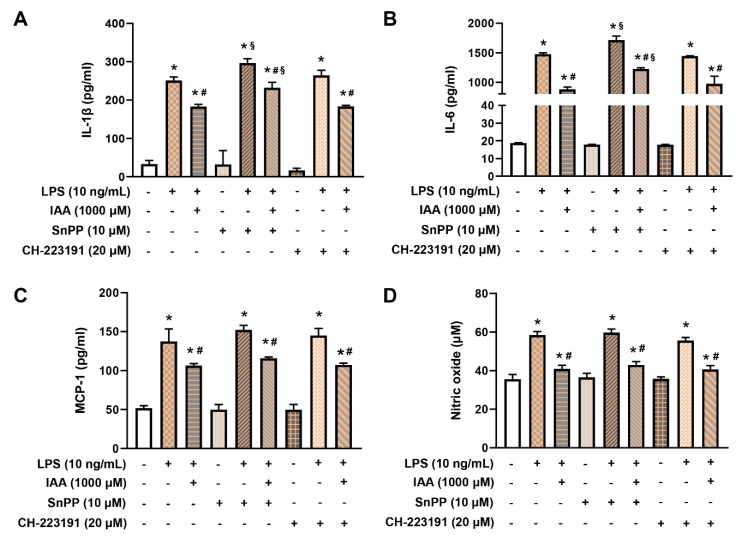
Effect of SnPP and CH-223191 on the protective action of IAA on generation of IL-1β, IL-6, MCP-1, and NO induced by LPS in RAW264.7 cells. The supernatants of cultured RAW264.7 were subjected to analysis of (**A**) IL-1β, (**B**) IL-6, (**C**) MCP-1, and (**D**) NO content after pretreatment with IAA (1000 μM) plus SnPP (10 μM) or CH-223191 (20 μM) for 12 h followed by LPS (10 ng/mL) exposure for 16 h in the presence or absence of IAA and SnPP/CH-223191. Results are given as mean ± SEM. *n* = 3. * *p* < 0.05 versus control group. ^#^ *p* < 0.05 versus LPS, LPS + SnPP, or LPS + CH-223191 group. ^§^ *p* < 0.05 versus corresponding control group treated with LPS or LPS + IAA without SnPP and CH-223191. SnPP, tin protoporphyrin IX; NO, nitric oxide; IAA, indole-3-acetic acid; LPS, lipopolysaccharide.

To determine whether HO-1 mediates the protective effects of IAA against LPS-induced proinflammatory response, we used tin protoporphyrin IX (SnPP), a potent inhibitor of HO-1 activity. As shown in Figure 6A,B, the elevation in levels of IL-1β and IL-6 induced by LPS were augmented by SnPP (*p* < 0.05). Notably, exposure of cells to SnPP significantly impaired the alleviative effects of IAA on secretion of IL-1β and IL-6 caused by LPS stimulation. For MCP-1, no significant effects were found between cells exposed to IAA LPS or LPS + IAA following treatment with or without SnPP (Figure 6C). In order to examine whether the inhibitory effect of IAA on proinflammatory cytokines depends on aryl hydrocarbon receptor (AhR), an endogenous receptor for IAA, cells were treated with AhR antagonist CH-223191. Addition of CH-223191 did not alter the inhibitory effect of IAA on LPS-induced increment in levels of IL-1β, IL-6, and MCP-1. This result indicates that the alleviative effect of IAA on the expression of proinflammatory cytokines does not depend on AhR.

### 2.6. The Inhibitory Effect of IAA on the Production of ROS and NO is Independent of HO-1 Induction and AhR

**Figure 7:**
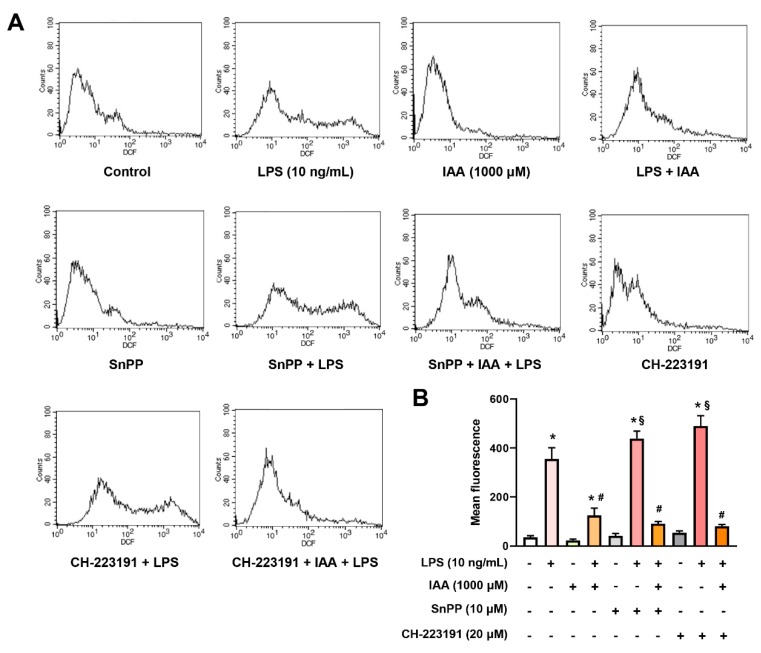
Effects of SnPP and CH-223191 on the scavenge of ROS by IAA. (**A**) Representative flow cytometry plots. (**B**) Statistical results presented by bar chart. Cells were treated with IAA (1000 μM) with or without SnPP (10 μM) or CH-223191 (20 μM) for 12 h, followed by LPS (10 ng/mL) exposure for 16 h in the presence or absence of IAA plus SnPP or CH-223191. DCFH-DA probe was added to detect the ROS level. Results are shown as mean ± SEM. *n* = 3. * *p* < 0.05 versus control group. ^#^ *p* < 0.05 versus LPS group. ^§^ *p* < 0.05 versus corresponding group without SnPP and CH-223191. DCFH-DA, 2′,7′-dichlorofluorescin diacetate; ROS, reactive oxidative species; SnPP, tin protoporphyrin IX; IAA, Indole-3-acetic acid; LPS, lipopolysaccharide.

To investigate whether the mitigation effect of IAA on ROS and NO relied on HO-1 or AhR, HO-1 inhibitor SnPP or AhR antagonist CH-223191 was added during the treatment. As illustrated in Figure 7A,B, treatment with SnPP or CH-223191 significantly potentiated ROS production in RAW264.7 exposed to LPS (*p* < 0.05). However, the protective role of IAA against LPS-induced ROS generation was not impeded by either SnPP or CH-223191. Additionally, blocking HO-1 or AhR showed no effect on LPS-induced NO accumulation and the suppression of NO by IAA (Figure 6D). These findings demonstrate that blockage of HO-1 or AhR is not sufficient to repress the protective role of IAA against LPS-induced production of ROS and NO.

## 3. Discussion

IAA, one of the key auxin class plant hormones, is critical for many crucial physiological processes [16,17]. Importantly, IAA has been found to be synthesized by gut microbiota from dietary tryptophan [18]. Previous studies with cells and mice have revealed the anti-oxidative and anti-inflammation effects of IAA [11,19,20]. The present study reveals that IAA suppresses LPS-induced inflammatory stress and free radical generation in RAW264.7 macrophages. This conclusion is supported by following observations. (1) LPS-triggered production of proinflammatory cytokines and free radicals was reduced by IAA treatment. (2) IAA increased the expression of HO-1. (3) Inhibition of HO-1 activity blocked the effect of IAA on LPS-induced elevation in IL-1β and IL-6, while demonstrated no effect on ROS and NO generation. (4) The inhibitory action of IAA on the LPS-induced release of proinflammatory cytokines (IL-1β and IL-6) and free radical (NO and ROS) was unaltered by antagonism of AhR receptor.

It has been widely known that cytokines play a key role in manipulating immune responses [21]. Following the stimulation of LPS, multiple proinflammatory cytokines, which can accelerate inflammatory responses, are secreted by macrophages [6]. Accordingly, the effect of IAA on the expression and secretion of proinflammatory factors induced by LPS was determined. We observed that IAA suppressed the secretion of proinflammatory cytokines such as IL-1β, IL-6, and MCP-1, suggesting a potential anti-inflammatory activity of IAA. Consistently, studies by Krishnan et al. demonstrated that IAA significantly mitigates increases in IL-1β, TNF-α, and MCP-1 mRNA levels in macrophages treated with palmitates and LPS [19]. NF-κB is a multimeric complex transcription factor that regulates the expression of a large array of genes involved in inflammation [22]. This complex is retained in inactive form in the cytoplasm through binding to inhibitors called IκB proteins in unstimulated cells [23]. IκB is phosphorylated and undergoes degradation by ubiquitination upon exposure to LPS, leading to release of free NF-κB p65, which then enters into the nucleus to activate the transcription of inflammatory cytokines [24]. Our study revealed that IAA inhibited LPS-induced translocation of NF-κB p65 from the cytoplasm to the nucleus, thereby is in favor of relieving the inflammatory responses.

Apart from functioning as modulators of the inflammatory reactions, proinflammatory cytokines also facilitate the induction of free radicals such as NO and ROS [25]. iNOS catalyzes the production of NO which is a messenger molecule involved in the pathogenesis of numerous inflammatory diseases [26]. In our study, we observed that IAA suppressed the expression of iNOS and thus the production of NO in RAW264.7 macrophages. Although moderate ROS is beneficial to host defense, excessive ROS generated during acute and chronic inflammation may lead to oxidative damage, lipid peroxidation, DNA fragmentation, and protein carbonylation [27]. In particular, ROS generated by macrophages stimulated by LPS may cause excessive inflammatory reactions, leading to tissue damage [28]. IAA was previously reported to exhibit free radical elimination effects [29,30]. Consistently, we found that treatment of macrophages with IAA obviously blocked the generation of ROS induced by LPS.

HO-1 is one of the key detoxification enzymes that plays a crucial role in the prevention of oxidative and inflammatory injury [31]. Previous results from soybean plants have reported that the resistance of IAA on oxidative stress caused by drought depends on the induction of HO-1 [32]. On the other hand, HO-1 induction relies on the production of NO. Our study extends this observation to mammalian cells. In murine macrophages, IAA also induced an increase in the level of HO-1. Excessive NO generation induced by LPS stimulation is known as proinflammatory mediator [33]. Nevertheless, IAA itself did not affect the synthesis of NO in RAW264.7 macrophages. In line with our results, IAA-induced increase in HO-1 has also been reported in other cell model. For example, a study conducted on human dental pulp stem cells have shown that IAA induces HO-1 to resist oxidative stress induced by hydrogen peroxide [20]. Our study indicates that IAA also increases the expression of HO-1 in the context of inflammation stimulation. Since HO-1 functions as an enzyme with both anti-oxidant and anti-inflammatory activity [31], it is worth exploring whether the beneficial effects of IAA depends on HO-1. For this goal, SnPP was applied to intervene the activity of HO-1. We found that the suppression on LPS-induced expression of proinflammatory factors IL-1β and IL-6 by IAA was HO-1 dependent, whereas the inhibition of HO-1 activity did not abolish the scavenging effect of IAA on free radical. Consistently, an early study has shown that IAA is capable of scavenging free radical directly [29]. IAA has been identified as one of the ligands of AhR [34,35]. However, our results demonstrated that IAA reduces free radical and proinflammatory factor production induced by LPS in a manner that is independent of AhR in macrophages. Consistent with our results, AhR-independent suppressive role on proinflammatory cytokines has also been confirmed for other AhR ligands (indole) in dendritic cells [36].

## 4. Materials and Methods

### 4.1. Materials

Indole-3-acetic acid and LPS (lipopolysaccharides from *Escherichia coli* O55:B5) were obtained from Sigma-Aldrich (St. Louis, MO, USA). Tin protoporphyrin IX (SnPP) was obtained from Cayman Chemical (Ann Arbor, MI, USA). CH-223191 was purchased from MedChem Express (Monmouth Junction, NJ, USA). Dulbecco’s modified Eagle’s medium (DMEM) was from Biological Industries (Kibbutz Beit Haemek, Israel). Fetal bovine serum (FBS) was purchased from Hyclone (South Logan, UT, USA). Cell counting kit (CCK-8), TRIzol RNA extraction reagent, nitric oxide assay kit, 2′,7′-dichlorofluorescin diacetate (DCFH-DA), and Hoechst 33,342 were obtained from Applygen Technologies Inc. (Beijing, China). Hieff^®^ qPCR SYBR^®^ Green Master Mix and Hifair^®^ II 1st Strand cDNA Synthesis SuperMix for qPCR (gDNA digester plus) were procured from Yeasen biotech Co. (Shanghai, China). Antibody against β-actin was from Santa Cruz Bio. (Santa Cruz, CA, USA). Anti-HO-1 was purchased from ABclonal Inc. (College Park, MD, USA). Anti-NF-κB p65 was from Beyotime (Shanghai, China). ECL Western blotting detection kit, horseradish peroxidase (HRP)-conjugated anti-rabbit immunoglobulin G (IgG), and anti-mouse IgG were purchased from Huaxingbio. (Beijing, China). ELISA kits for IL-1β, IL-6, TNF-α, and MCP-1 were bought from ExCell Biotech (Taicang, Jiangsu, China).

### 4.2. Cell Culture and Cell Viability

RAW264.7 cells, a murine macrophage cell line, were procured from American-Type Culture Collection (ATCC, Rockville, MD, USA) and were grown in Dulbecco’s modified Eagle’s medium (DMEM) supplemented with 10% fetal bovine serum (FBS). Cells were maintained in an incubator containing 5% CO_2_ with humidified atmosphere at 37 °C. To assess the cell viability of RAW264.7 cells in response to IAA. Cells were seeded in a 96-well plate at a density of 1 × 10^4^ cells per well and were treated with 0, 25, 50, 100, 150, 250, 500, 750, and 1000 μM of IAA in DMEM medium supplemented with 5% FBS, followed by addition of CCK-8 reagent (1:10 diluted in medium) and an incubation for 40 min at 37 °C in cell incubator. The absorbance at 450 nm was measured by using a microplate reader (Bio-Rad, Hercules, CA, USA). The cell viability relative to control was calculated according to the following formula: (A_sample_ − A_blank_)/(A_control_ − A_blank_) × 100.

### 4.3. RNA Extraction and Real-Time PCR (RT-qPCR)

Total RNA was extracted using commercial TRIzol reagent (Applygen Technologies Inc., Beijing, China) following the protocol provided by manufacturer. In brief, cells obtained from each well were lysed with 1 mL of TRIzol, followed by addition of 0.2 mL of chloroform. Upon hand-shaking for 15 s and letting stand on ice for 5 min, the lysates were centrifuged for 15 min at 12,000 g at 4 °C. The aqueous phase was moved into a new tube and mix with isopropanol, after which RNA pellets were obtained by centrifugation and washed with 75% ethanol in DEPC water. The pellets were then dissolved in DEPC-treated water. The yield and purity of each RNA sample were determined by Nanodrop OneC (Thermo Scientific, Waltham, MA, USA). To obtain cDNA, 2 μg of RNA was subjected to DNAase digestion and reverse transcription performed using Hifair^®^ II 1st Strand cDNA Synthesis SuperMix for qPCR according to the user’s manual. Subsequently, PCR amplification was conducted by a mixture containing Hieff^®^ qPCR SYBR^®^ Green Master Mix, forward primer, reverse primer, template cDNA, and sterile ultra-pure water. The primer sequences for each gene were listed in Table 1. The relative expression level of target gene was quantified by 2^−ΔΔCT^ method [37] taking β-actin as reference.

### 4.4. Enzyme-Linked Immunosorbent Assay

Double-antibody sandwich enzyme-linked immunosorbent assays (ELISAs) were performed to measure the levels of IL-1β, IL-6, TNF-α, and MCP-1 in culture medium. Briefly, samples/standards and biotinylated primary antibodies were added to monoclonal antibody-coated wells to form an immune complex. Following washing of the plates, horseradish peroxidase-labeled avidin was added to bind to the biotin. After washing, substrate solution and stop solution were added respectively. Optical density (OD) values were measured at 450 nm using a microplate reader (Bio-Rad, Hercules, CA, USA).

### 4.5. Nitric Oxide Measurement

NO content in cell culture medium was determined using a commercial NO Detection Kit. According to the protocols supplied with assay kit, 50 μL of cell culture medium or NaNO_2_ standards were mixed with an equal volume of Griess reagent I. After a 5 min incubation, 50 μL of Griess reagent II was added to each well, followed by determination of OD value at 490 nm. The content of NO was calculated with reference to a standard curve of NaNO_2_.

### 4.6. Reactive Oxygen Species Determination

The intracellular ROS content was evaluated by DCFH-DA probe according to the instruction provided by manufacturer. In brief, the cells after drug treatment were exposed to 10 µM DCFH-DA incubation in DMEM for 30 min at 37 °C, followed by washing three times with DMEM. The intracellular ROS was observed under a fluorescence microscope, after which cells were collected and subjected to more accurately analysis using a flow cytometer (BD Biosciences, San Jose, CA, USA).

### 4.7. Immunofluorescence

After drug exposure, cells were washed with PBS and then were fixed with freshly-prepared 4% paraformaldehyde in PBS for 15 min at room temperature. Following permeabilization using 1% Triton X-100 in PBS for 10 min on ice, samples were subjected to an 1 h blocking with 1% goat serum in PBS at room temperature. Then, they were incubated with a 1:200 dilution of primary antibodies against NF-κB p65 overnight at 4 °C, after which cells were exposed to PBS three times and an 1 h incubation with fluorescein isothiocyanate (FITC)-conjugated secondary antibodies in 1:100 dilution in 1% goat serum at room temperature in the dark. A total of 0.01 mg/mL of Hoechst 33,342 was added to stain the nucleus. Images were immediately captured by using a microscope equipped with a U-RFL-T fluorescence microscopy unit (Olympus, Tokyo, Japan).

### 4.8. Protein Extraction and Western Blot

Whole-cell lysates were prepared using radioimmunoprecipitation assay (RIPA) lysis buffer containing mixed inhibitors of protease and phosphatase on ice for 30 min. Lysates were then centrifuged to obtain supernatants at 12,000 g for 30 min at 4 °C. Bicinchoninic acid (BCA) assay was used to determine protein concentrations. Each sample containing 20 μg of protein was separated by 12% sodium dodecyl sulfate-polyacrylamide gel electrophoresis (SDS-PAGE) and subsequently transferred onto polyvinylidene fluoride (PVDF) membranes. After blocking with 5% skim milk in tris-buffered saline with tween (TBST) for 1 h at room temperature, membranes were incubated with primary antibodies (1:1000 dilution) overnight at 4 °C. Then the membrane was rinsed with TBST buffer, followed by an incubation for 1 h at room temperature with horseradish peroxidase (HRP)-labeled secondary IgG antibodies (1:5000 dilution) corresponding to the origin of primary antibody. After incubation with enhanced chemiluminescence (ECL) substrate, the bands were visualized by chemiluminescence imaging system (MiniChemi, Beijing, China) and quantified using Image J software (National Institutes of Health, Bethesda, MD, USA).

### 4.9. Statistical Analysis

All values are indicated as means  ±  standard error of the mean (SEM). The statistical analysis was performed by one-way ANOVA followed by Tukey’s multiple comparison using GraphPad Prism version 8.0 (GraphPad Software Inc., CA, USA). Differences between groups with *p*-value less than 0.05 were regarded as statistically significant.

## 5. Conclusions

Taken together, the present study reveals that IAA exhibits anti-inflammatory and anti-oxidative activity in LPS-stimulated RAW264.7 cells. In response to LPS, the secretion of proinflammatory cytokines IL-1β and IL-6 was attenuated by IAA treatment, which is partially attributed to the induction of HO-1. In addition, the scavenging role of IAA on free radicals (NO and ROS) generated from macrophages exposed to LPS relied on neither HO-1 nor AhR. These findings imply that IAA is a potential gut microbial metabolite critical for maintaining the redox and immune homeostasis. Macrophages play an essential role in inflammatory diseases pertaining to bacterial LPS. Our findings offer some valuable and new insights into the molecular mechanism of IAA against proinflammatory response induced by LPS in macrophages. Future studies are required to further enrich and elucidate the mechanism of IAA in amelioration of inflammatory diseases. Moreover, manipulation of IAA-producing gut microbiota via nutritional strategies may provide novel target to improve human health.

## Figures and Tables

**Table 1:** Sequences of primers used for RT-qPCR.

Gene	Accession No.	Forward Primer (5’-3’)	Reverse Primer (5’-3’)
*MCP-1*	NM_011333.3	TCCCAATGAGTAGGCTGGAG	TCTGGACCCATTCCTTCTTG
*IL-1β*	XM_006498795.4	AGGCTCCGAGATGAACAACA	TTGTCGTTGCTTGGTTCTCC
*IL-6*	NM_031168.2	TAGTCCTTCCTACCCCAATTTCC	TTGGTCCTTAGCCACTCCTTC
*TNF-α*	NM_013693.3	GCTGAGCTCAAACCCTGGTA	AGTACTTGGGCAGATTGACCT
*iNOS*	NM_010927.4	CAACGTGAAGAAAACCCCTTGT	AACATTCTGTGCTGTCCCAGT
*HO-1*	NM_010442.2	CACGCATATACCCGCTACCT	CCAGAGTGTTCATTCGAGCA
*β-actin*	NM_007393.5	GTGGGAATGGGTCAGAAGGA	CTTCTCCATGTCGTCCCAGT

## Abbreviations

AhR	aryl hydrocarbon receptor;
DMEM	Dulbecco’s modified Eagle’s medium;
FBS	fetal bovine serum;
FITC	fluorescein isothiocyanate;
IL-1β	interleukin-1β;
IL-6	interleukin-6;
HO-1	heme oxygenase-1;
IAA	indole-3-acetic acid;
iNOS	inducible nitric oxide synthase;
LPS	lipopolysaccharide;
MCP-1	monocyte chemoattractant protein-1;
NF-κB	nuclear factor kappa B;
NO	nitric oxide;
ROS	reactive oxidative species;
SnPP	tin protoporphyrin IX;
TNF-α	tumor necrosis factor-α.
